# Supplementary material for: Cognitive Stimulation and Its Effects on Well-Being, Executive Functions, and Brain-Derived Neurotrophic Factor in Older Adults from a Mexican Geriatric Center: A Quasi-Experimental Study
Source: Nurs Rep. 2025 Apr 30;15(5):151. doi: 10.3390/nursrep15050151 (PMC12113664; doi:10.3390/nursrep15050151)
Supplement: Supplementary file 1 [file nursrep-15-00151-s001.zip › nursrep-3525323-Supplementary Material File S1.docx]

File S1. Example of a Level 1 Exercise for Each of the 28 Activities

**Cuadernillo de estimulación cognitiva**

Resumen del cuadernillo a manera de ejemplo, debido a

que su extensión supera las 800 hojas.

**INTRODUCCIÓN**

Este cuadernillo de estimulación cognitiva ha sido diseñado especialmente para adultos mayores, con el objetivo de fortalecer y mantener activas las funciones cognitivas clave para un envejecimiento saludable: **flexibilidad cognitiva, control inhibitorio, memoria de trabajo, planificación, razonamiento, atención y concentración y velocidad de procesamiento**.

La estimulación y el mantenimiento de estas funciones son esenciales, ya que permiten a los adultos mayores enfrentar los desafíos de la vida diaria con mayor independencia, seguridad y satisfacción.

El **razonamiento** es fundamental para la resolución de problemas y la toma de decisiones, ayudando a los adultos mayores a gestionar situaciones cotidianas de manera eficiente. La **atención**, por su parte, permite concentrarse en las tareas y minimizar distracciones, lo cual es crucial para realizar actividades de manera efectiva y sin errores. La **flexibilidad cognitiva** es la capacidad de adaptarse a situaciones nuevas o cambiantes, ajustando el pensamiento de manera eficiente.La **memoria de trabajo** permite mantener y manipular información en la mente a corto plazo, lo que es esencial para muchas actividades diarias. El **control inhibitorio** ayuda a regular respuestas impulsivas y a enfocarse en tareas relevantes. La **planificación** permite organizar actividades y establecer estrategias para alcanzar objetivos. Finalmente, la **concentración y velocidad de procesamiento** optimizan el desempeño cognitivo al mejorar el enfoque y la rapidez en el procesamiento de la información.

La falta de estimulación de estas funciones puede llevar a un deterioro cognitivo progresivo, afectando la calidad de vida y la independencia del adulto mayor. Para evitar estas consecuencias, este cuadernillo incluye ejercicios distintos para cada una de las funciones mencionadas, cada uno de ellos en tres niveles de dificultad, para facilitar una práctica progresiva y adaptada a las habilidades individuales. A través de estos ejercicios, se busca que el adulto mayor no solo mantenga sus capacidades cognitivas, sino que también experimente una mayor facilidad y confianza al realizar actividades diarias, contribuyendo a su bienestar y calidad de vida.

Este cuadernillo representa un compromiso hacia la búsqueda de un envejecimiento saludable, digno y pleno para cada adulto mayor. Cada actividad aquí contenida ha sido pensada con dedicación, reconociendo el valor de la experiencia, la historia y la fortaleza de quienes han recorrido largos caminos. Con estos ejercicios, deseamos acompañarlos en una etapa que puede estar llena de aprendizajes y satisfacciones, promoviendo un bienestar que trascienda lo físico y fortalezca lo mental y emocional.

**Que este cuadernillo sea una herramienta para continuar descubriendo, creando y viviendo cada día con entusiasmo y claridad.**

**NIVEL 1**

**ACTIVIDAD 1**

**Analogías verbales**

**Ejercicio 1.1**

**Instrucciones:** Resuelve cada analogía eligiendo la palabra que mejor complete la relación y escríbela en la línea correspondiente. Tienes 5 minutos para completar todas las analogías. ¡Buena suerte!

**Leche es a vaca como lana es a… O .**

**Blanco es a negro como el día es a la… N .**

**León es a la selva como tiburón es a… M .**

**Cucaracha es al insecto como rosa es a… F .**

**Lagrima es a tristeza como sonrisa a… F .**

**ACTIVIDAD 2**

**Crucigrama**

**Ejercicio 2.1**

**Instrucciones:**  Completa el crucigrama llenando todas las casillas en blanco. Dispones de 5 minutos para responder. ¡Buena suerte!.

**ACTIVIDAD 3**

**Secuencia de figuras**

**Ejercicio 3.1**

**Instrucciones:** A continuación, dibuja las tres figuras que siguen en la secuencia. Tienes 3 minutos para completar esta tarea. No te preocupes por los detalles; lo más importante es seguir la secuencia y disfrutar el momento.


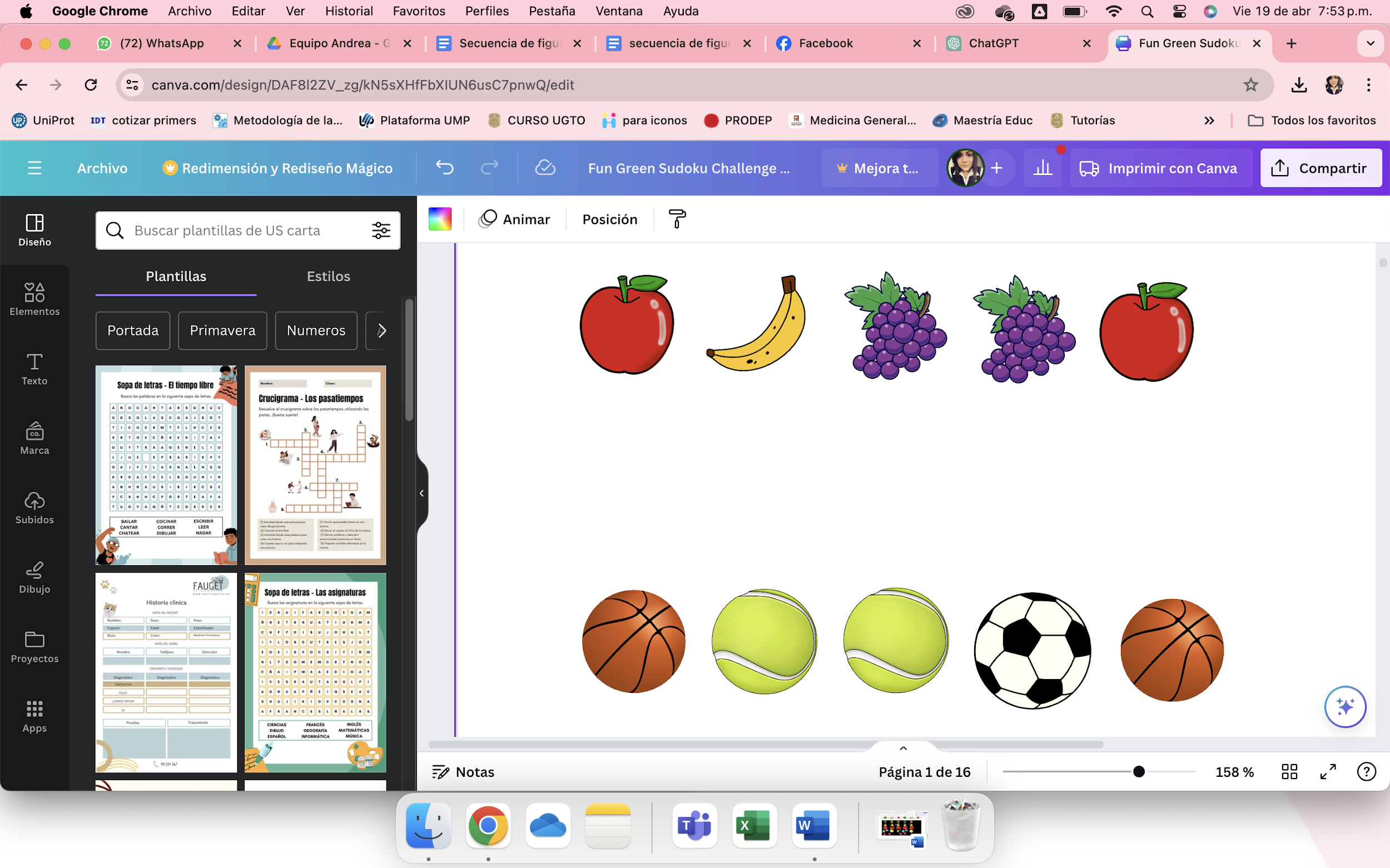

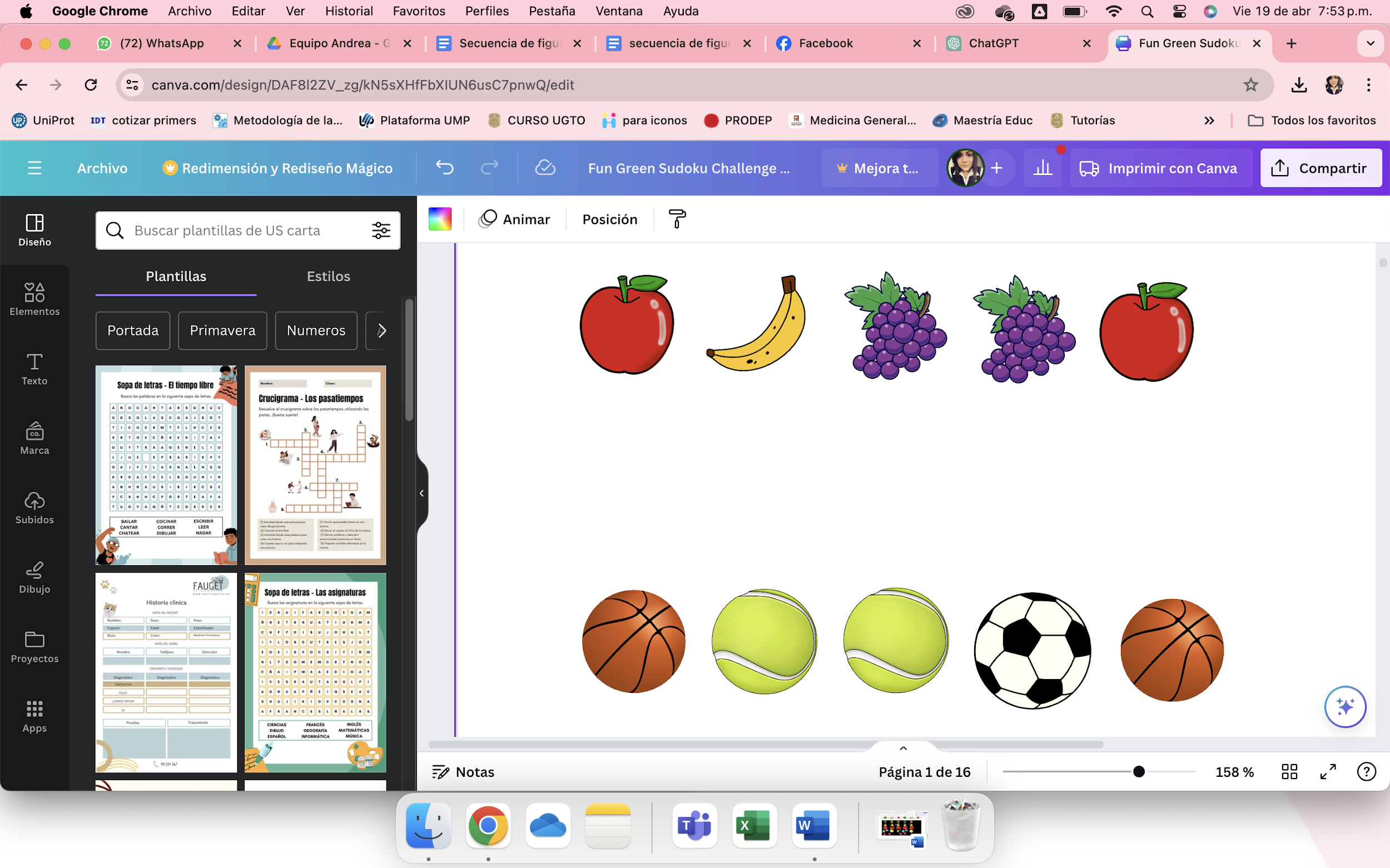


**ACTIVIDAD 4**

**Secuencia de números**

**Ejercicio 4.1**

**Instrucciones:** A continuación, escribe las cuatro sucesiones correspondientes a cada una de las secuencias numéricas que se te presentan. Dispondrás de 12 minutos para completar todas las secuencias. Tómate tu tiempo y asegúrate de seguir el patrón. ¡Buena suerte!.

- **31, 33, 35, 37, 39, 41… ________________________________**
- **43, 46, 49, 52, 55, 58… ________________________________**
- **87, 95, 103, 111, 119, 127…_____________________________**
- **36, 41, 46, 51, 56, 61…_________________________________**
- **7, 16, 25, 34, 43, 52…__________________________________**
- **75, 79, 83, 87, 91, 95..._________________________________**
- **17, 24, 31, 38, 45, 52…_________________________________**
- **52, 58, 64, 70, 76, 82…_________________________________**

**ACTIVIDAD 5**

**Sopa de letras**

**Ejercicio 5.1**

**Instrucciones:** Encuentra y marca todas las palabras escondidas en la sopa de letras, que están listadas en la parte inferior. Las palabras pueden estar en diferentes direcciones. Tómate tu tiempo y disfruta de la actividad. ¡Diviértete!

**ACTIVIDAD 6**

**Enigmas**

**Ejercicio 6.1**

**Instrucciones:** Lee con atención la pregunta y completa la respuesta. Dispones de la letra inicial y una pista como ayuda.

| **Enigma**: ¿Qué es algo que cuanto más grande es, menos se ve?.  O______________________.  **Pista**: Es algo que puedes encontrar al aire libre, especialmente en la noche. |
| --- |
| **Enigma**: Soy ligero como una pluma, pero ni el hombre más fuerte del mundo puede sostenerme por mucho tiempo. ¿Qué soy?    R______________________.  **Pista**: Es algo que haces cada segundo. |
| **Enigma**: Tengo ciudades, pero no casas. Tengo montañas, pero no árboles. Tengo agua, pero no peces. ¿Qué soy?  M_______________________.  **Pista**: Lo utilizas para orientarte. |

**ACTIVIDAD 7**

**Las compras**

**Ejercicio 7.1**

**Instrucciones:** Lee con atención cada ejercicio y resuelvelo.

1. **¿Cuál es el resultado de la suma de 18 + 12?.** R= ______
2. **Si tienes 80 manzanas y regalas 20, ¿cuántas manzanas te quedan?.** R= __
3. **¿Cuál es el resultado de 5 x 6?.** R= ______
4. **Si tienes 24 caramelos y los repartes en 6 bolsas, ¿cuántos caramelos hay en cada bolsa?.** R= ______
5. **María tiene 50 pesos. Si gasta 20 pesos en un libro, ¿cuánto dinero le queda?.** R= ______
6. **En un parque hay 10 árboles y 4 de ellos son frutales. ¿Cuántos no son frutales?.** R= ______

**Actividad 7.2**

**Instrucciones:** Lee con atención cada ejercicio y resuelvelo.

1. **¿Cuál es el resultado de la suma de 22 + 16?**. R= ______
2. **Si tienes 90 manzanas y decides regalar 25, ¿cuántas manzanas te quedan?**. R= ______
3. **¿Cuál es el resultado de 4 x 7?**. R= ______
4. **Si tienes 36 caramelos y los distribuyes en 9 bolsas, ¿cuántos caramelos hay en cada bolsa?**. R= ______
5. **Ana tiene 80 pesos. Si gasta 30 pesos en una película, ¿cuánto dinero le queda?**. R= ______
6. **En un jardín hay 12 árboles y 6 de ellos son manzanos. ¿Cuántos no son manzanos?**. R= ______

**ACTIVIDAD 8**

**Replica**

**Ejercicio 8.1**

**Instrucciones:** Tienes 30 segundos para observar detenidamente la imagen del lado izquierdo; te recomendamos utilizar un cronómetro para medir el tiempo. Una vez que haya transcurrido el tiempo, cubre la imagen del lado izquierdo con la lámina de ocultación y procede a replicar la imagen en el lado derecho, basándote en lo que recuerdas.

**ACTIVIDAD 9**

**La sombra**

**Ejercicio 9.1**

**Instrucciones:** Identifica cuál de las sombras del lado izquierdo corresponde al objeto del lado derecho y traza una línea para unirlos.

**ACTIVIDAD 10**

**Los laberintos**

**Ejercicio 10.1**

**Instrucciones:** Resuelve el laberinto, partiendo del letrero de inicio hasta llegar a la medalla. No debes levantar el lápiz de la hoja en ningún momento. Dispones de 1 minuto para completar el recorrido, comenzando desde la parte superior y avanzando hacia abajo. Recuerda que no podrás ver el camino antes de dibujarlo.

**ACTIVIDAD 11**

**Calculando**

**Ejercicio 11.1**

**Instrucciones:** Calcula la distancia en centimetros entre las dos figuras indicadas. Una vez que termines usa una regla para confirmar la distancia.

**ACTIVIDAD 12**

**¿Cuál es igual?**

**Ejercicio 12.1**

**Instrucciones:** Observa atentamente las figuras, identifica la figura que tiene el mismo tamaño que la primera y enciérrala con un círculo.

**
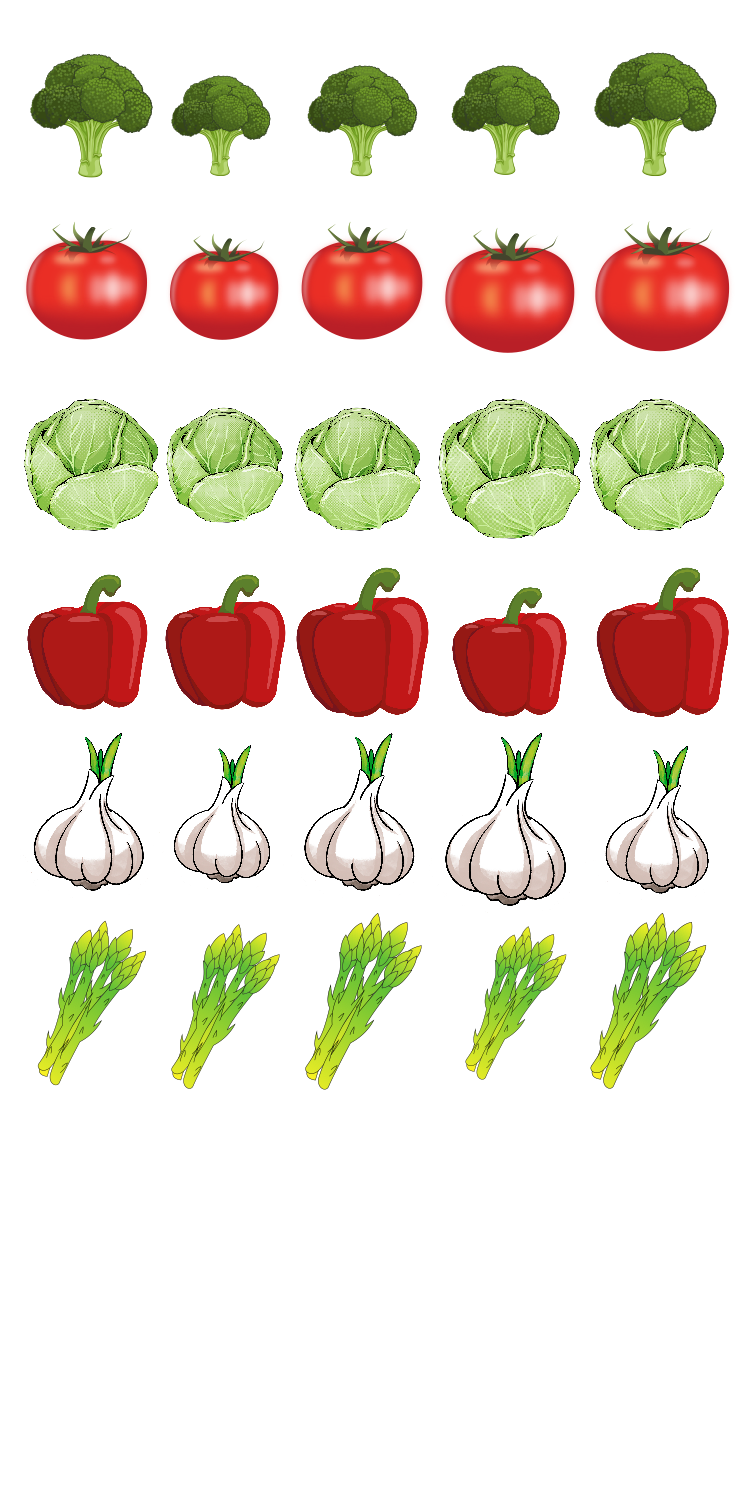
**

**ACTIVIDAD 13**

**La cuadricula**

**Ejercicio 13.1**

**Instrucciones:** Observa detenidamente la primera cuadrícula y presta atención a la ubicación de los puntos. Sin cubrir la primera cuadrícula, tacha ahora los números que corresponden a los espacios donde estaban los círculos.

| **•** |  |  |
| --- | --- | --- |
|  | **•** |  |
| **•** |  |  |

| **1** | **2** | **3** |
| --- | --- | --- |
| **4** | **5** | **6** |
| **7** | **8** | **9** |

**ACTIVIDAD 14**

**Ubícate**

**Ejercicio 14.1**

**Instrucciones:** Relaciona con flechas cada palabra del lado izquierdo con la figura correspondiente del lado derecho. ¿En qué lugar está la nube?.

La nube está arriba de la cruz.

La nube está de lado inferior derecho del rectángulo.

La nube está fuera del rectángulo de lado izquierdo.

La nube está en el centro del rectángulo.

La nube está de lado izquierdo de la cruz.

**ACTIVIDAD 15**

**Replica la imagen**

**Ejercicio 15.1**

**Instrucciones:** Primero, dedica un minuto a observar atentamente las figuras que se presentan, utilizando un cronómetro para controlar el tiempo. Una vez transcurrido el minuto, cubre la imagen con tu lámina de ocultación y comienza a dibujar las figuras que logres recordar. Asegúrate de que tus dibujos mantengan el mismo tamaño, posición y color que las originales. Si alguna figura es oscura, usa un lápiz para rellenarla.

Replica aquí la imagen.

**ACTIVIDAD 16**

**Pareja de imágenes**

**Ejercicio 16.1**

**Instrucciones:** Antes de comenzar, utiliza la lámina de ocultación para cubrir las imágenes que se encuentran en la parte inferior. Observa las siguientes parejas de figuras durante un minuto, utilizando un cronómetro para controlar el tiempo. Al finalizar el minuto, ahora cubre las parejas de imagenes con la lámina de ocultación y une cada pareja que recuerdes haber visto.


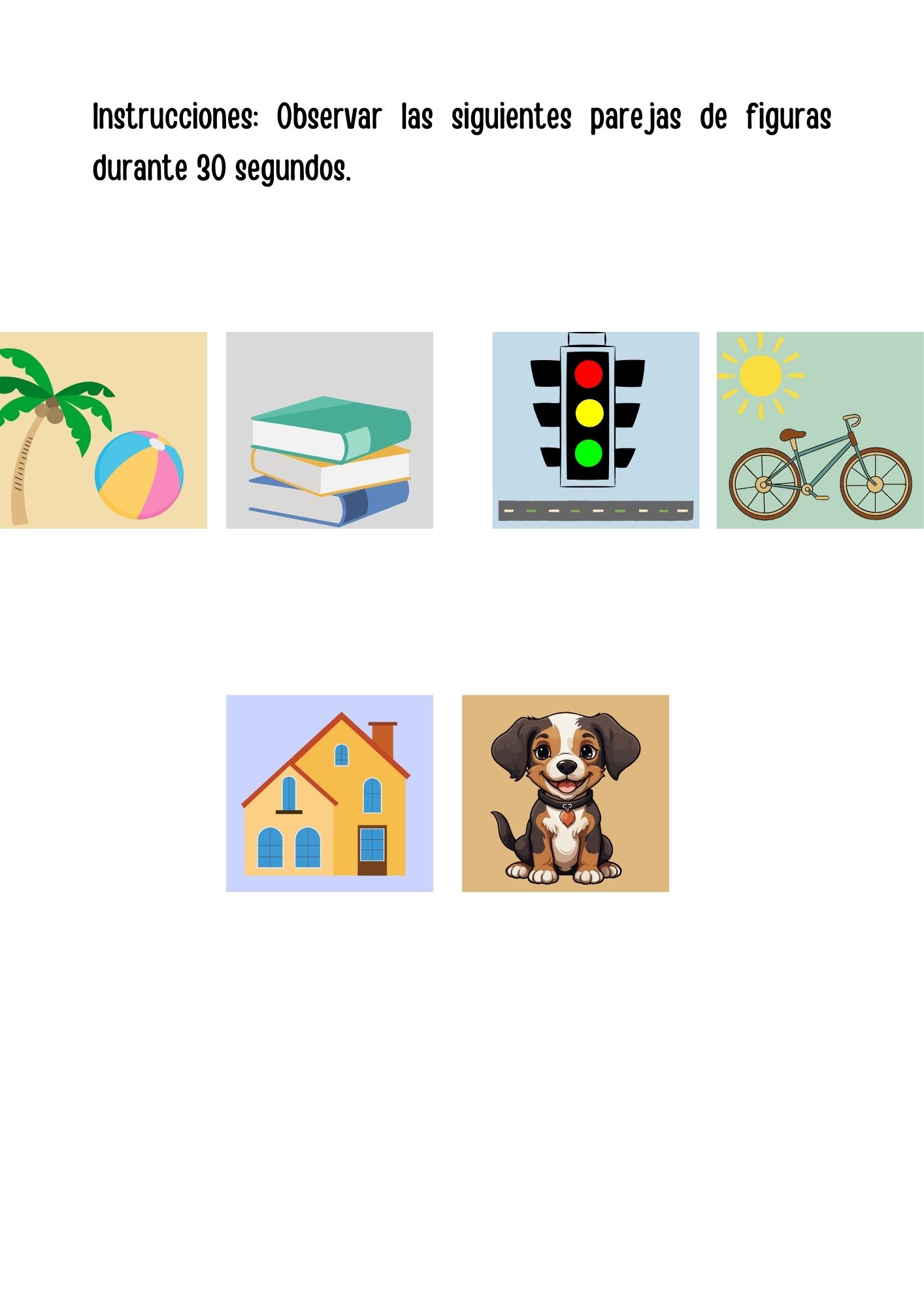


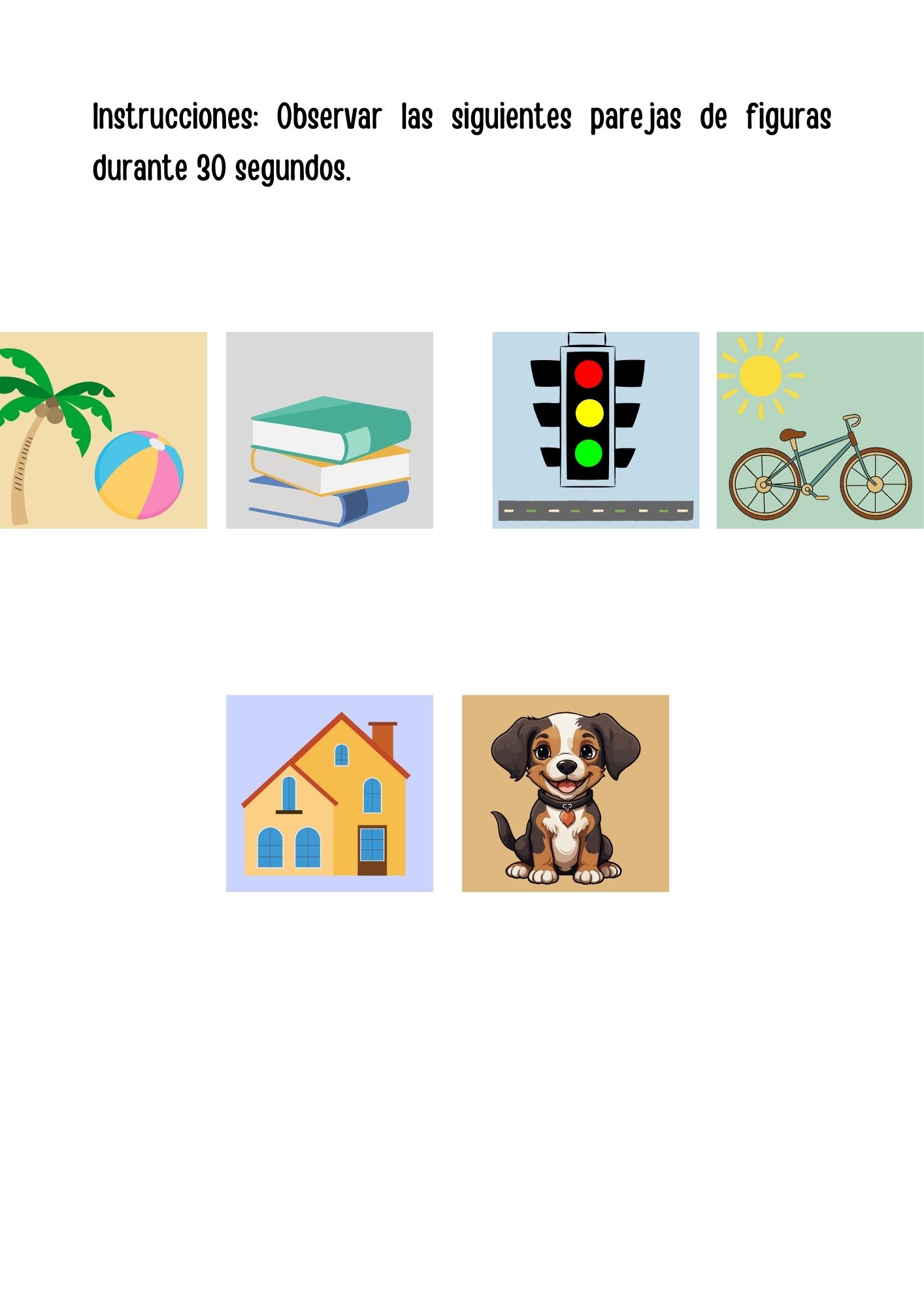


Une aquí cada pareja.


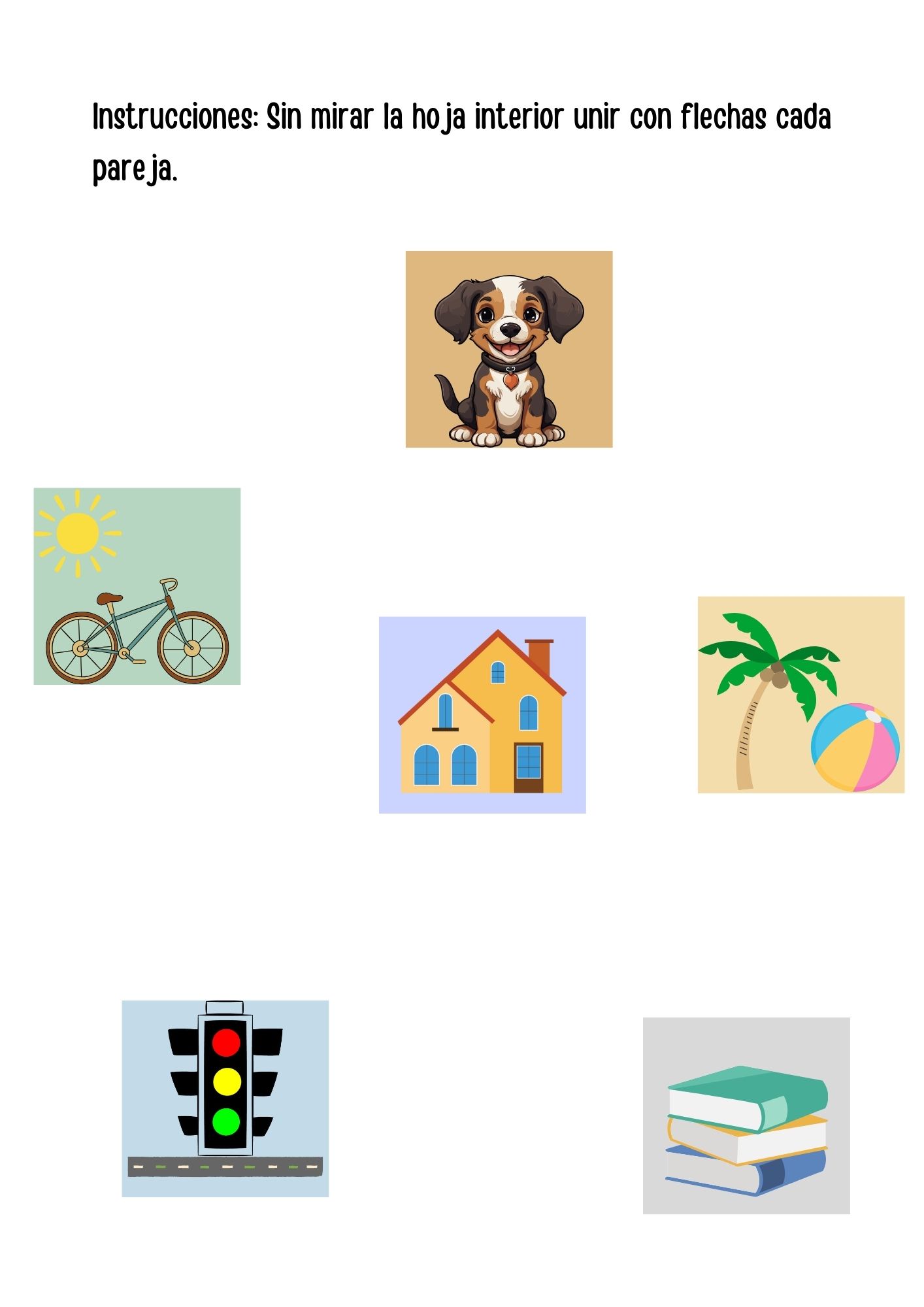

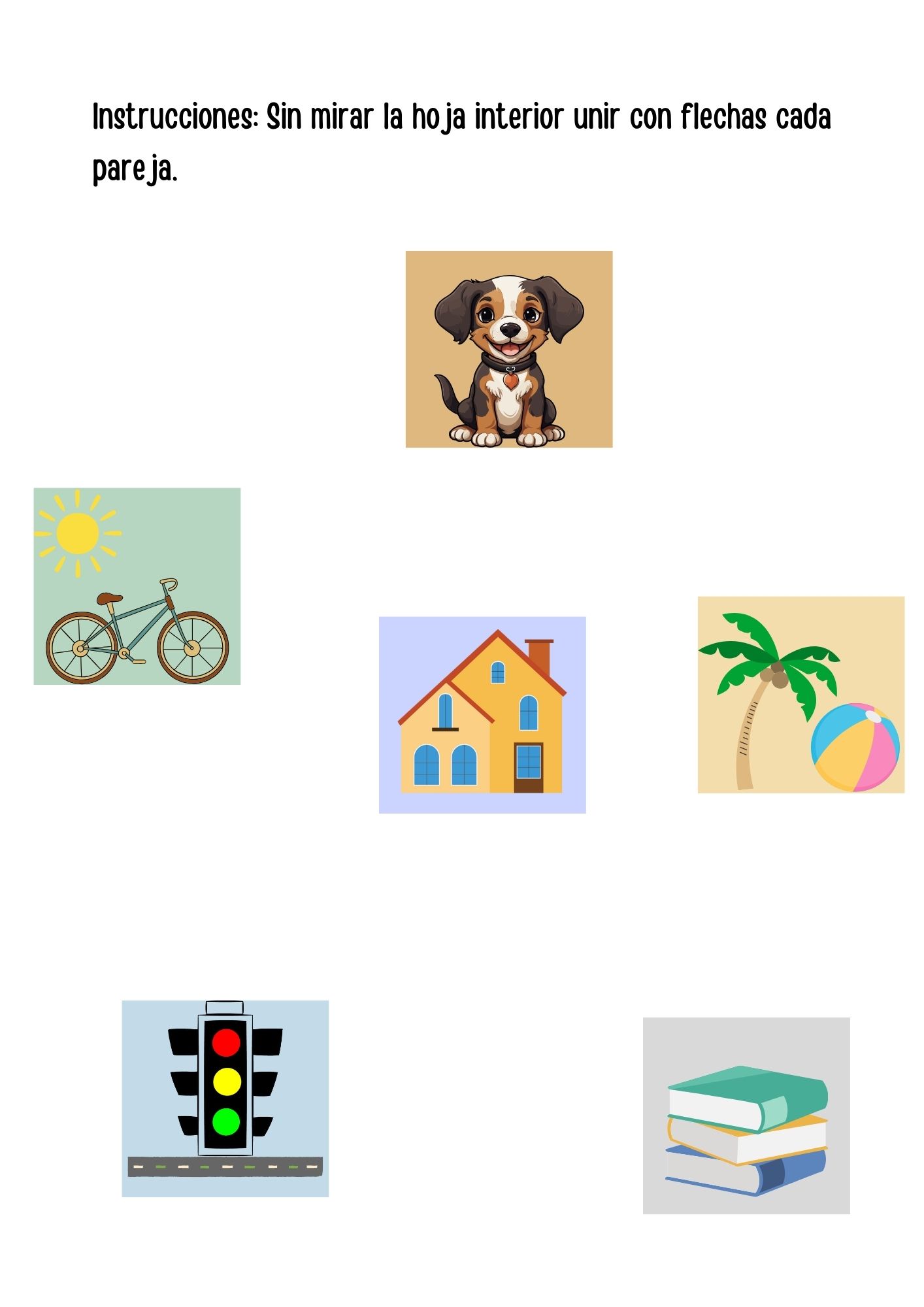

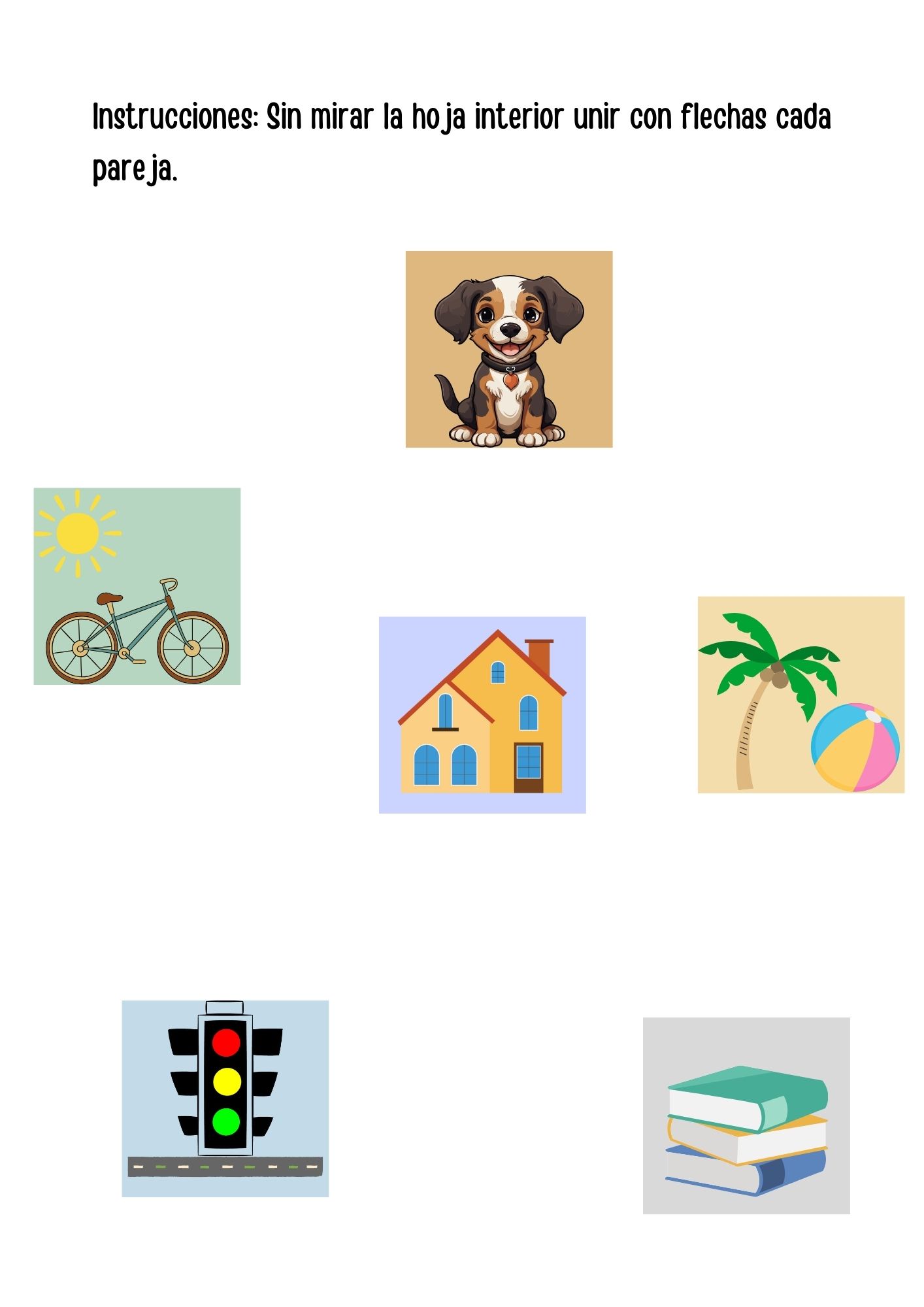


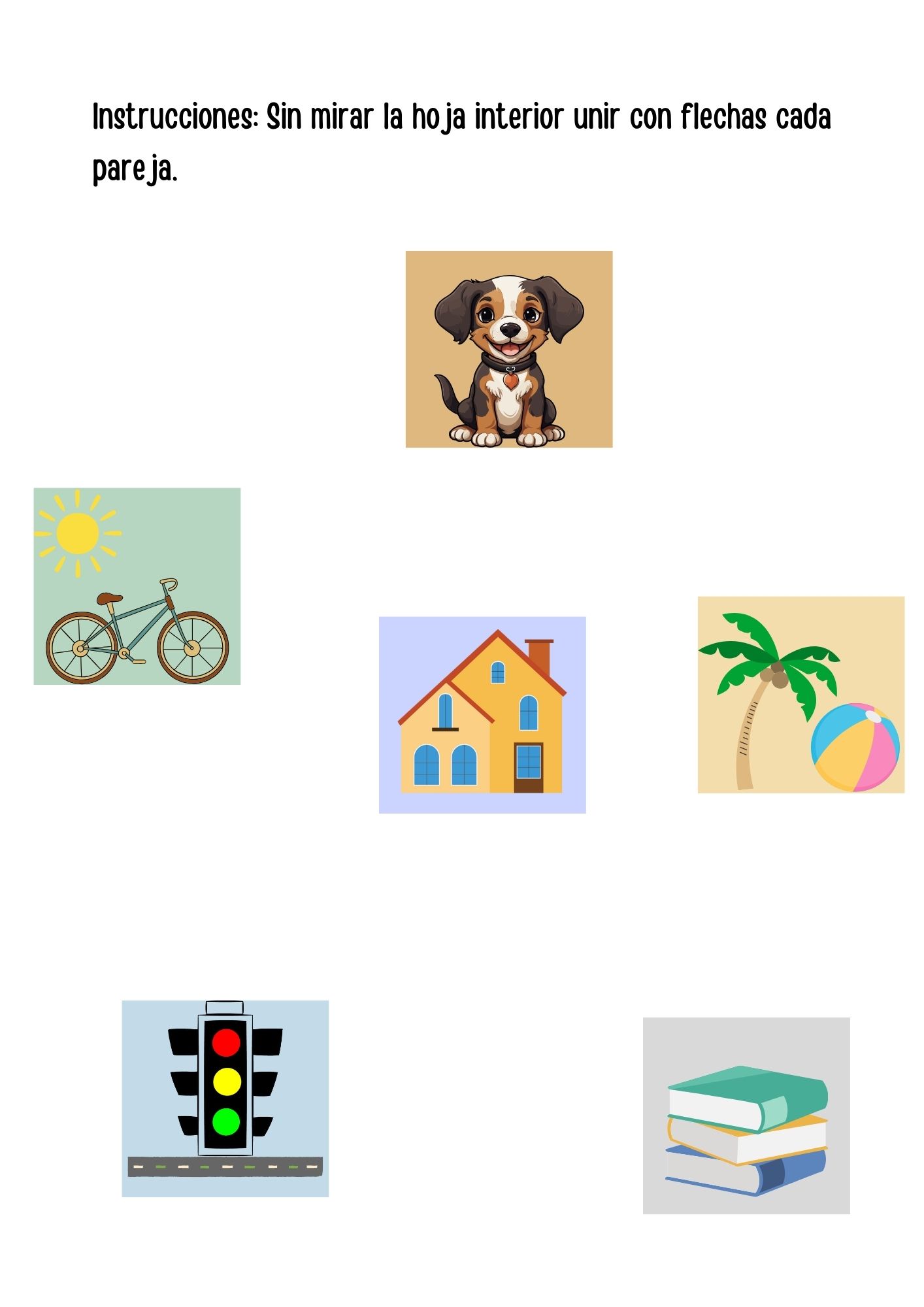


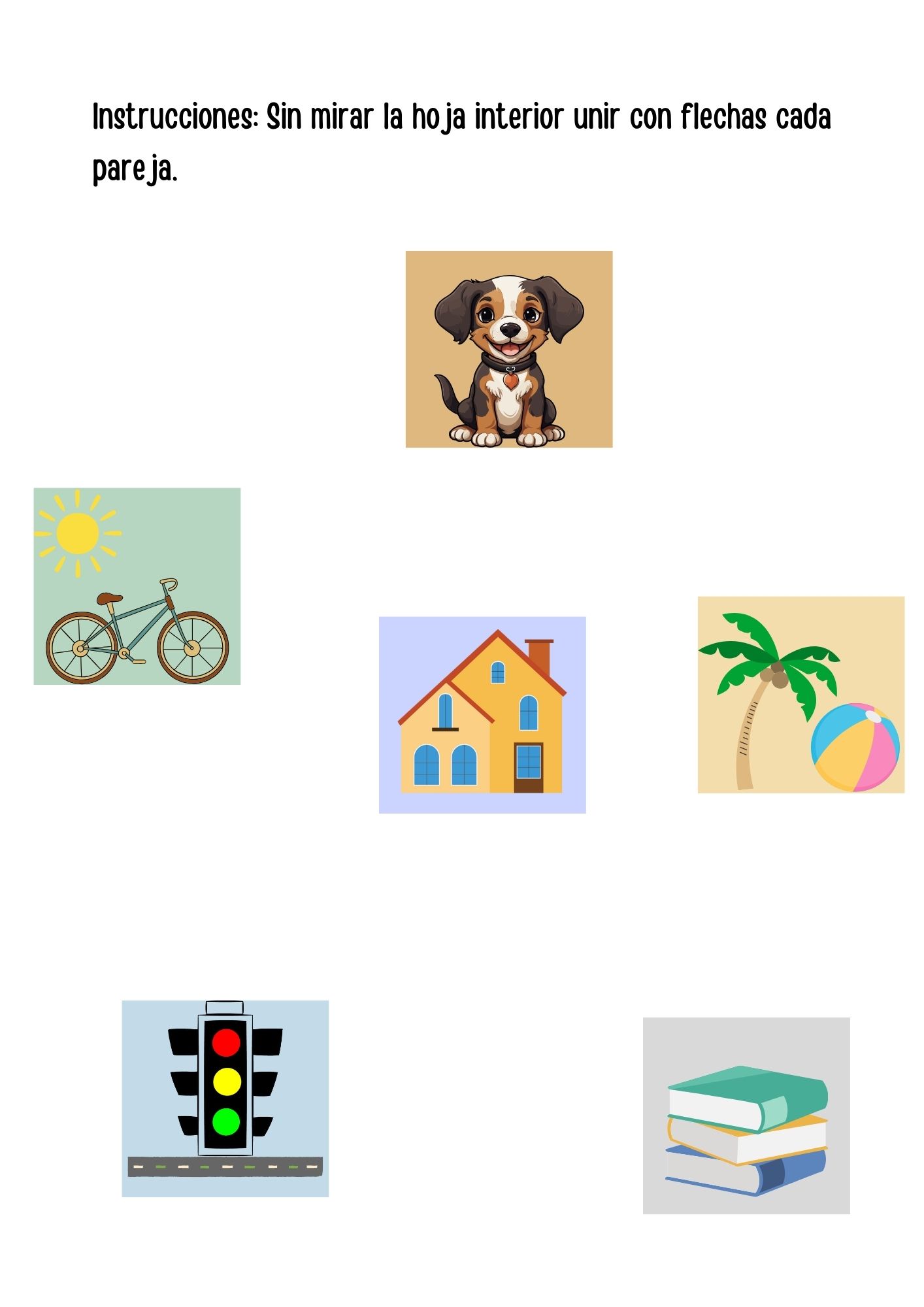

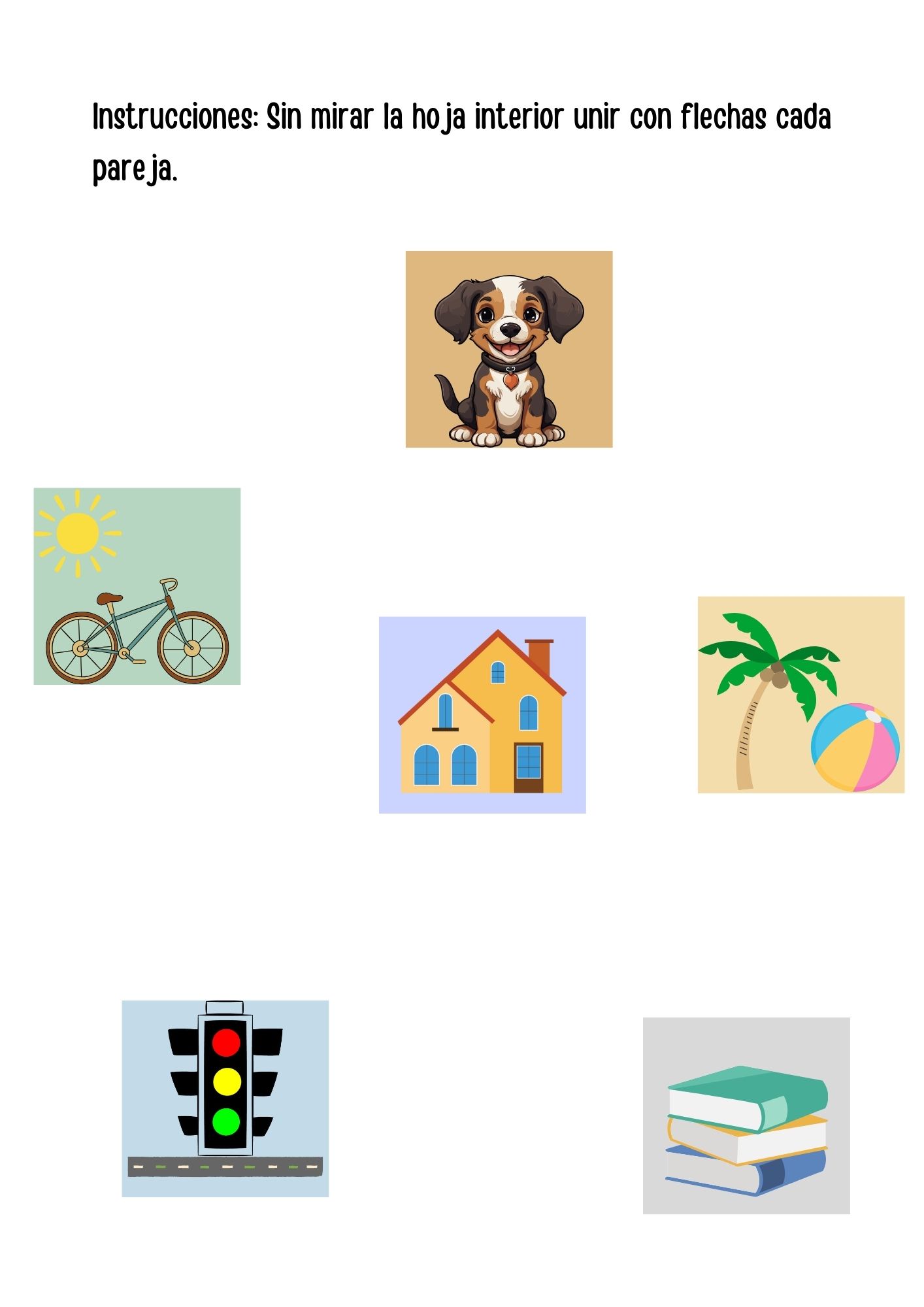


**ACTIVIDAD 17**

**Selección de imágenes**

**Ejercicio 17.1**

**Instrucciones:** Antes de comenzar, utiliza la lámina de ocultación para cubrir las imágenes que se encuentran en la parte inferior. Observa atentamente las figuras de la parte superior durante 30 segundos. Usa un cronómetro para medir el tiempo. Una vez transcurrido el tiempo, ahora cubre las imágenes superiores con la lámina de ocultación y encierra en un círculo las figuras que recuerdes haber visto.


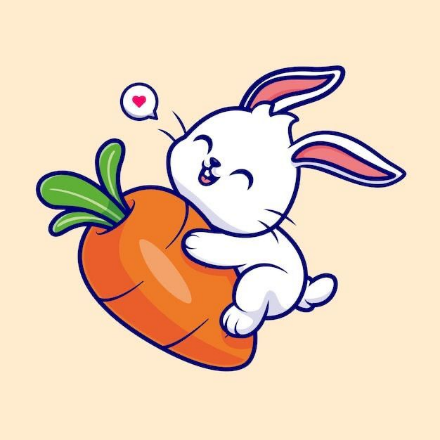

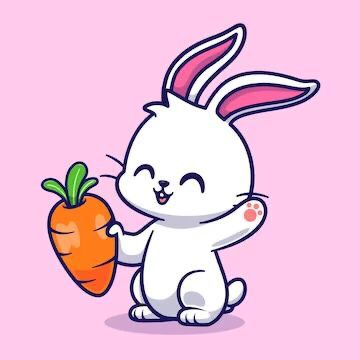

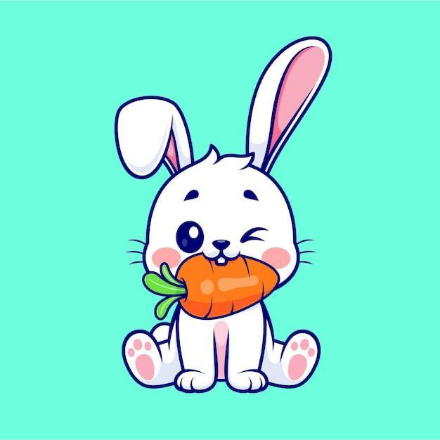


Circula aquí las figuras que recuerdes.


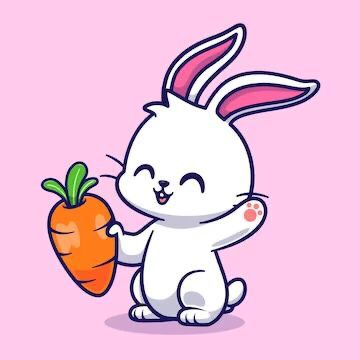

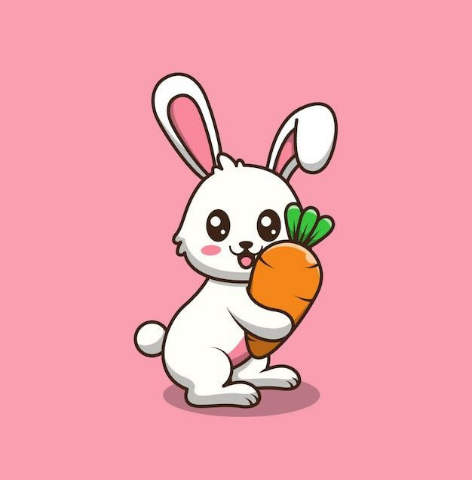

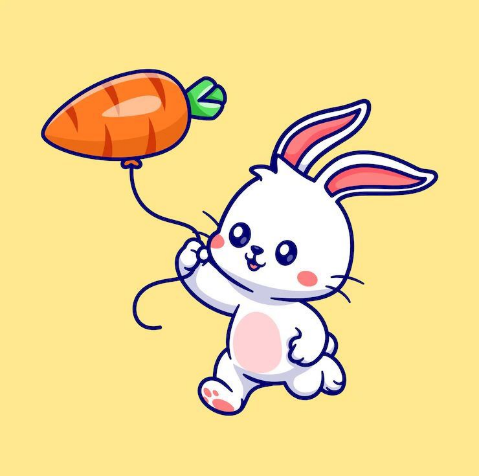

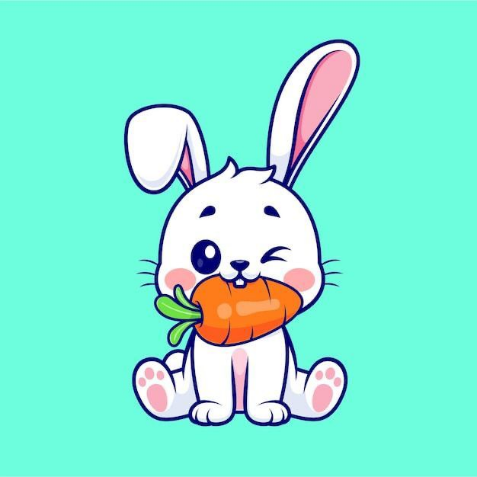

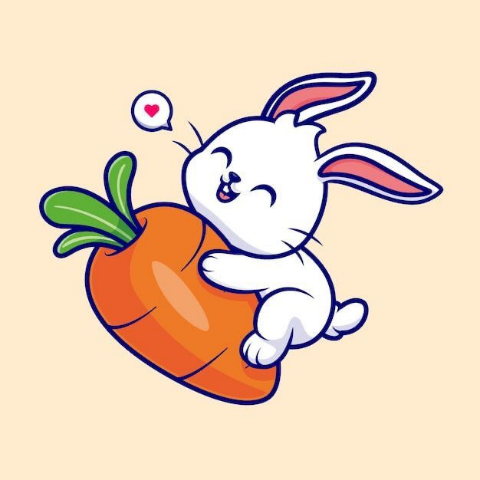

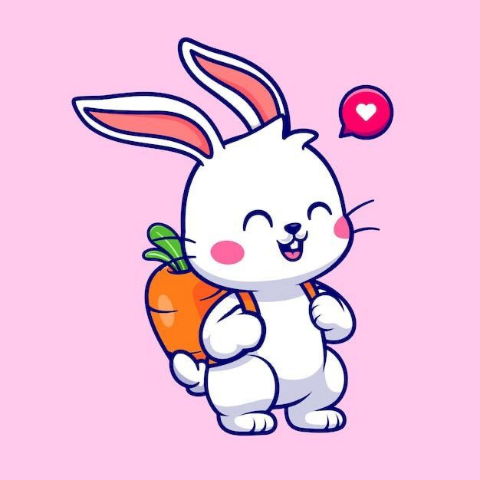


**ACTIVIDAD 18**

**Palabras**

**Ejercicio 18.1**

**Instrucciones:** Durante un minuto, observa atentamente las palabras y el color con el que están escritas. Utiliza un cronómetro para controlar el tiempo. Al finalizar el minuto, cubre las palabras con la lámina de ocultación. A continuación, escribe las palabras que recuerdes, asegurándote de utilizar también el mismo color con el que estaban escritas.

Gato

………………………………………….

………………………………………….

………………………………………….

………………………………………….

………………………………………….

Gorra

Jugo

Rojo

Lentes

**ACTIVIDAD 19**

**Formas y colores**

**Ejercicio 19.1**

**Instrucciones:** Observa atentamente las siguientes figuras y el color de cada una durante un minuto. Usa un cronómetro para medir el tiempo. Al finalizar, cubre las imágenes con una lámina de ocultación. Luego, de la serie de figuras que se encuentran abajo, colorea únicamente aquellas que estaban en la parte superior, respetando los colores originales.

**ACTIVIDAD 20**

**Dando orden**

**Ejercicio 20.1**

**Instrucciones:** Antes de comenzar, cubre las imágenes del lado derecho con la lámina de ocultación. Observa las imágenes y su número de orden durante un minuto, utilizando un cronómetro si lo deseas. Al finalizar el minuto, cubre las imágenes y ordénalas del lado izquierdo en el mismo orden en que estaban en el lado derecho


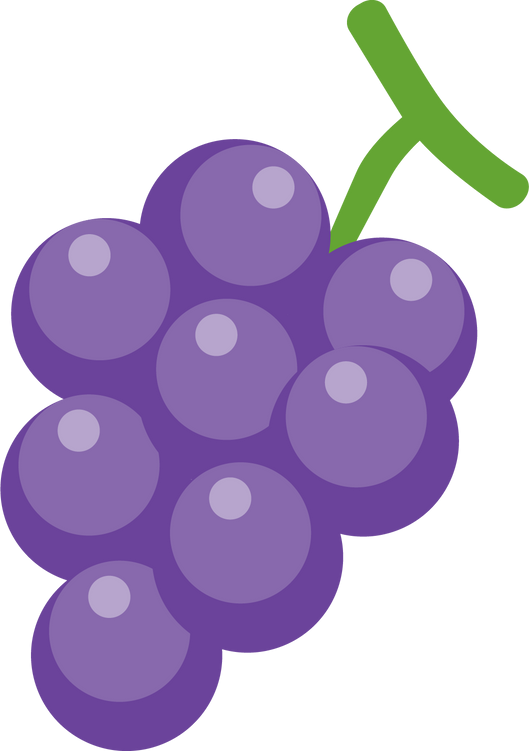


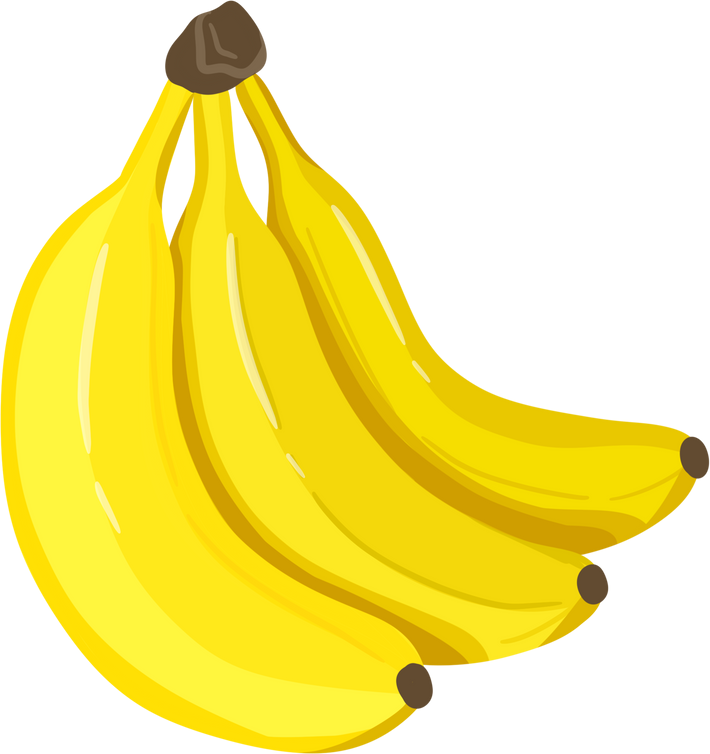

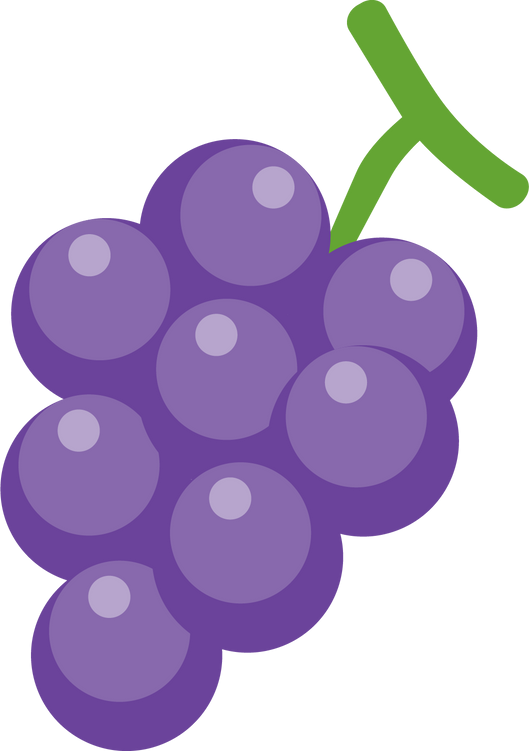

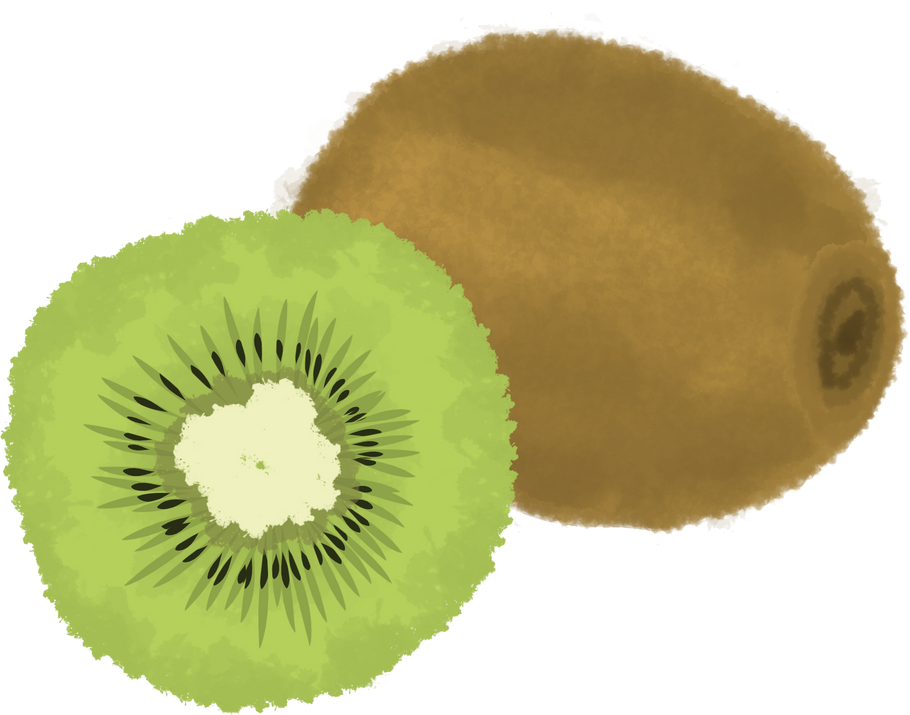


1


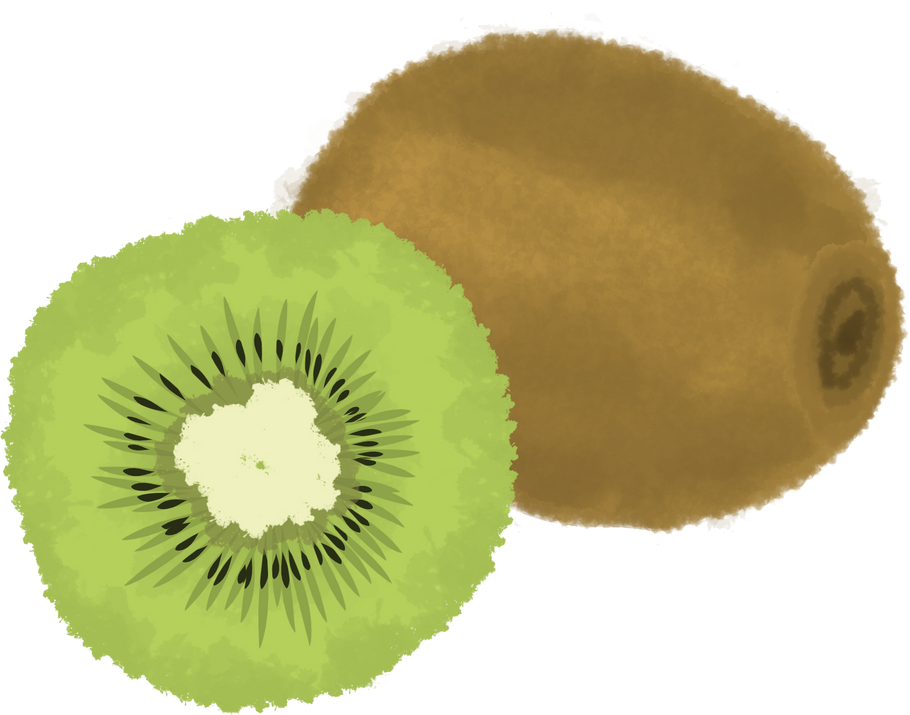

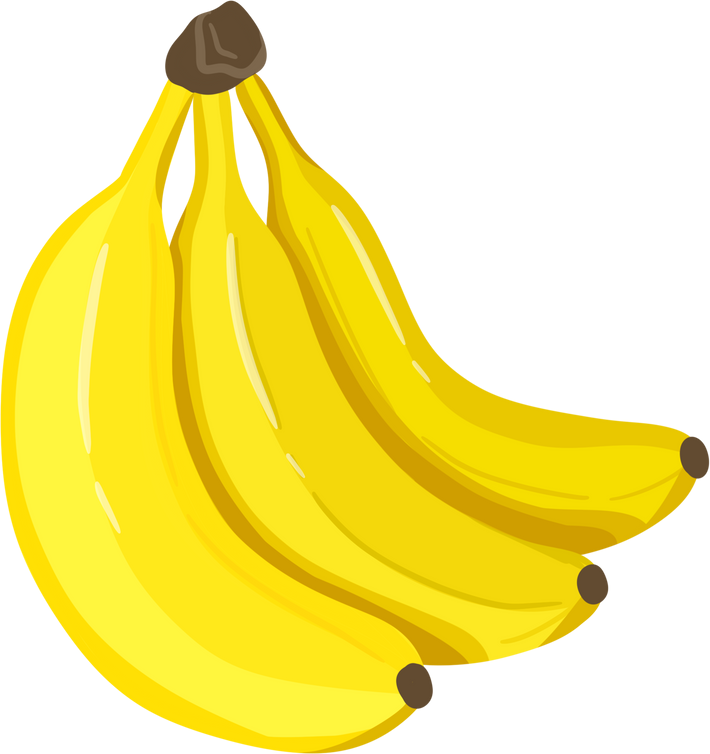

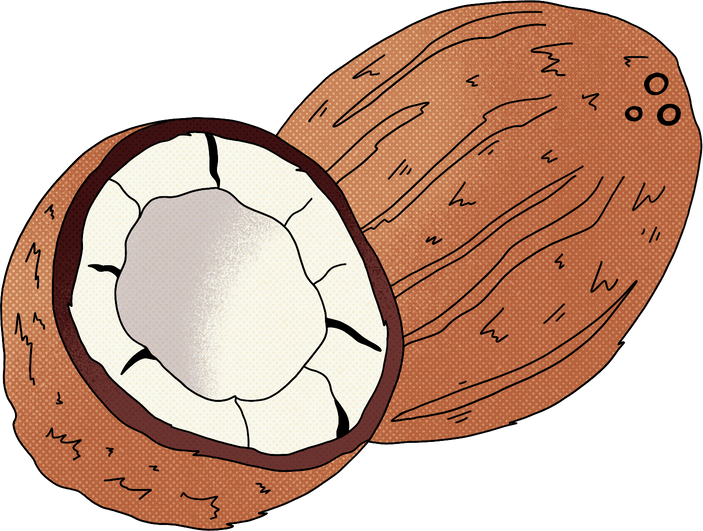

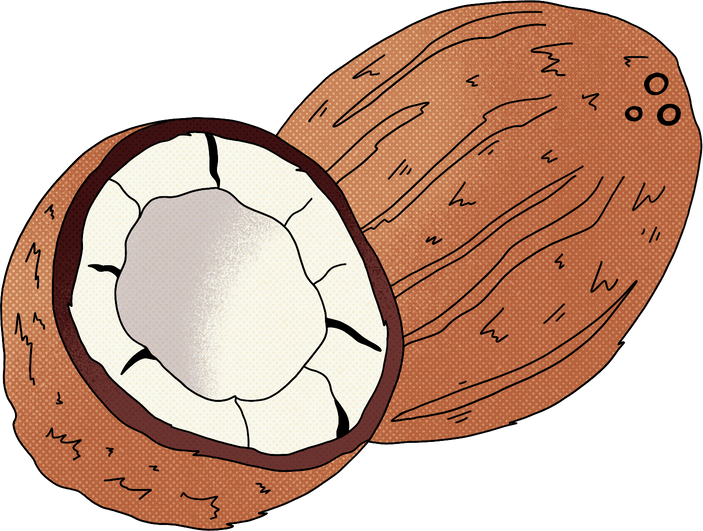


1

**ACTIVIDAD 21**

**¿Lo recuerdas?**

**Ejercicio 21.1**

**Instrucciones:** Antes de comenzar, cubre completamente las imágenes del lado derecho con la lámina de ocultación. Observa detenidamente la imagen del lado izquierdo durante un minuto, utilizando un cronómetro si lo consideras necesario. Al finalizar el tiempo, cubre la imagen del lado izquierdo y destapa la imagen del lado derecho. Verifica que ambas imágenes sean idénticas, y si la imagen del lado izquierdo carece de algún elemento presente en la del lado derecho, deberás agregarlo dibujándolo.


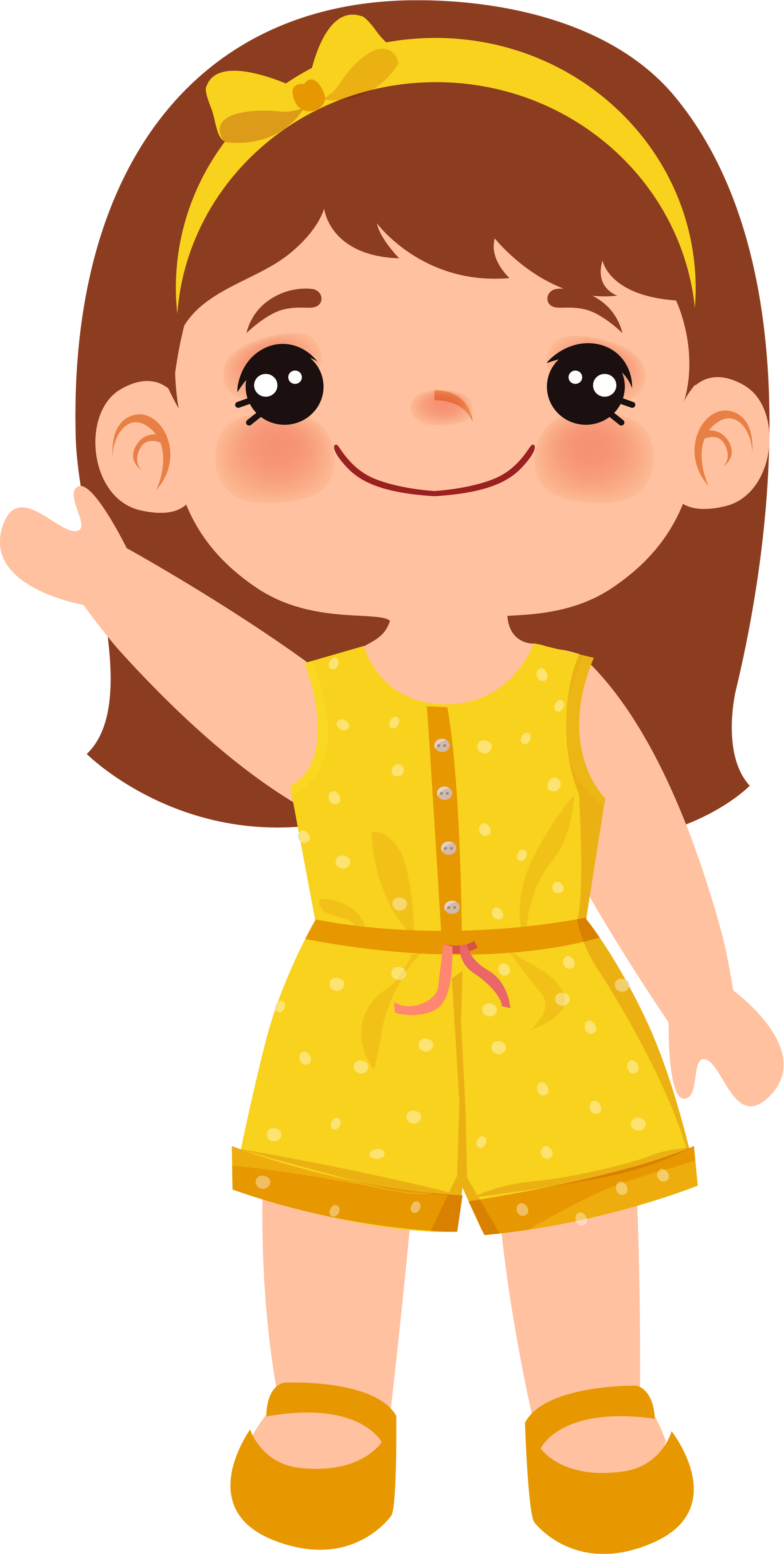


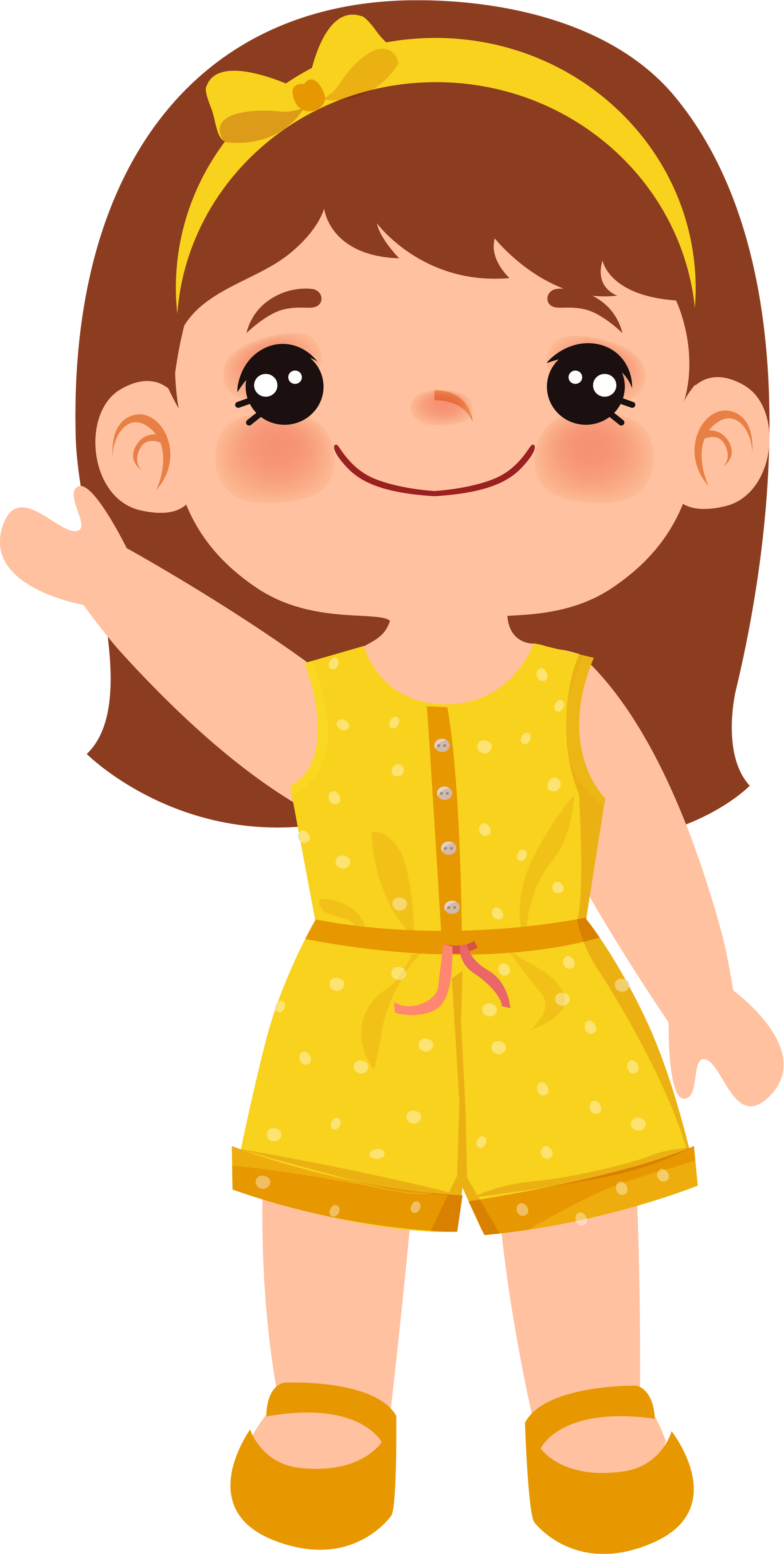

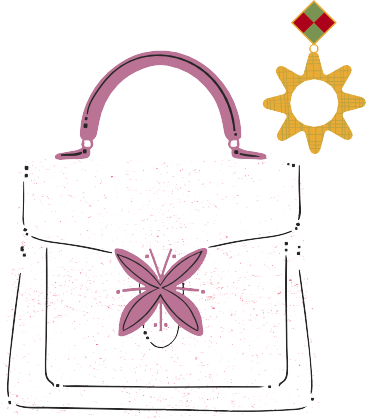

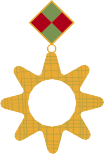

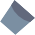

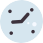


**ACTIVIDAD 22**

**Encuentra las diferencias**

**Ejercicio 22.1**

**Instrucciones:** Observa cuidadosamente las imágenes y circula las 5 diferencias.


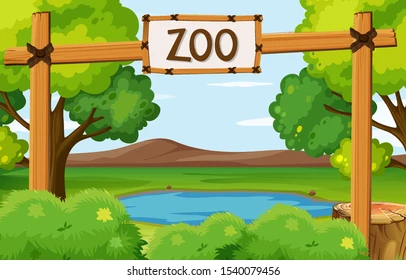

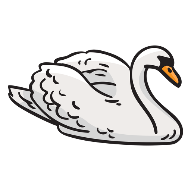

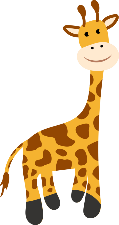

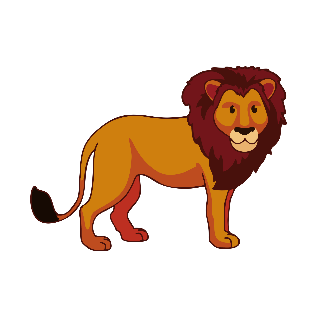

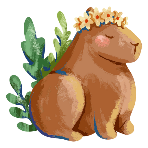

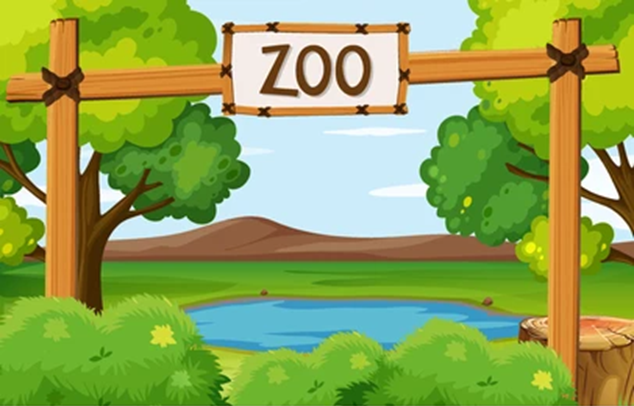

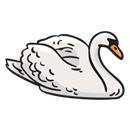

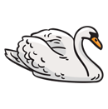

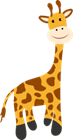

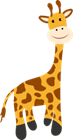

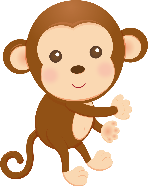

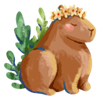

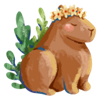


**ACTIVIDAD 23**

**Laberinto animado**

**Ejercicio 23.1**

**Instrucciones:** Guía al colibrí hasta la lavanda. Solo podrás avanzar en dirección horizontal o vertical si el color o la figura de la casilla siguiente es igual al de la casilla anterior. Intenta no levantar el lápiz y evita retroceder en tu recorrido.


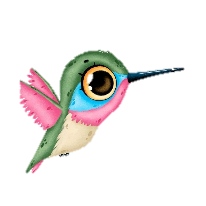

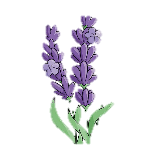

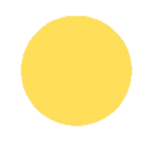

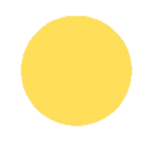

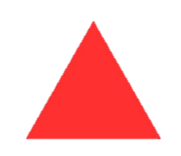

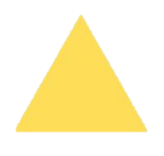

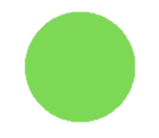

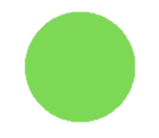

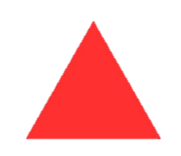

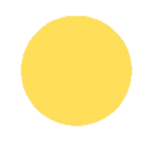

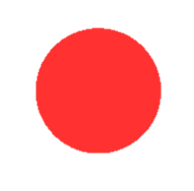

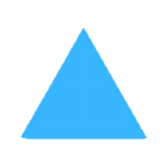

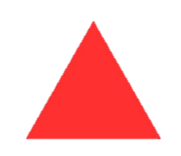

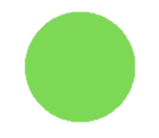

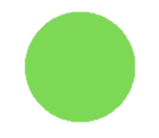


**ACTIVIDAD 24**

**Sodoku**

**Ejercicio 24.1**

**Instrucciones**: Completa el sudoku escribiendo los números del 1 al 4 en los espacios vacíos. Asegúrate de que no se repita ningún número en cada fila, columna y cuadrícula. ¡Diviértete resolviendo!.

| **4** |  | **1** | **3** |
| --- | --- | --- | --- |
|  | **1** |  |  |
| **2** |  | **3** | **1** |
|  |  |  | **2** |

**ACTIVIDAD 25**

**Conteo**

**Ejercicio 25.1**

**Instrucciones:** Coloca el número correspondiente debajo de cada símbolo según se indica. Si un símbolo no se menciona, se considera que equivale a 0. Luego, suma los valores de cada fila y, finalmente, calcula la suma total de los resultados obtenidos.

**🙍‍♀️=1 🙎=3 🙎‍♂️=2**

| 🙎 | 🙆‍♂️ | 🙎‍♂️ | 🙍‍♀️ |  |
| --- | --- | --- | --- | --- |
| + | + | + | = | + |
| 🙎‍♂️ | 🙍‍♀️ | 🙎 | 🙆‍♂️ |  |
| + | + | + | = | + |
| 🙍‍♀️ | 🙆‍♂️ | 🙎‍♂️ | 🙎 |  |
| + | + | + | = | = |
|  |  |  |  |  |

**ACTIVIDAD 26**

**Las vecinas**

**Ejercicio 26.1**

**Instrucciones:** Rodea con un círculo las letras que se encuentran dos posiciones antes de cada "W".

| P | J | U | H | W | N | B | E | K | E | W | I | T |
| --- | --- | --- | --- | --- | --- | --- | --- | --- | --- | --- | --- | --- |
| P | O | M | B | Y | S | W | M | U | Y | B | W | R |
| M | N | M | W | M | N | B | M | W | I | M | W | T |
| P | Ñ | E | N | U | T | E | N | B | Y | W | K | P |
| W | V | C | X | R | G | W | Y | B | V | W | Y | J |

**ACTIVIDAD 27**

**El cuento**

**Ejercicio 27.1**

**Instrucciones:** Lee con atención el cuento y luego responde las preguntas planteadas.

| **EL FARO DEL GUARDÍAN** |
| --- |
| En un pequeño pueblo costero, sobre los acantilados, se alzaba un faro antiguo conocido como "El Guardián de las Sombras". Los pescadores decían que su farero, aunque había muerto hacía muchos años, seguía vigilando en las noches de tormenta, guiando a los barcos perdidos entre las olas embravecidas.  Lucía, una joven del pueblo, había crecido escuchando esas historias. Su curiosidad la llevó a visitar el faro una noche especialmente oscura, cuando el viento y las olas rugían con fuerza. Subió por el empinado camino que llevaba a la torre y, al llegar, descubrió que la puerta estaba entreabierta, como si alguien la hubiera estado esperando.  Con nerviosismo, entró y subió la escalera de caracol hasta la cima. Allí, rodeada por el eco del mar y la luz giratoria, vio algo inesperado: una figura alta y delgada, vestida con ropa antigua, observando el horizonte. Era el farero, con una barba gris y un rostro pálido.  —¿Eres real? —preguntó Lucía, con el corazón acelerado.  —Soy el farero, aunque ya no pertenezco a este mundo —respondió él, con una voz suave que parecía fluir con el viento.  Le explicó que, muchos años atrás, había hecho un pacto con el mar para proteger las costas y los barcos que navegaban cerca. Aunque su cuerpo se había desvanecido con el tiempo, su sombra seguía atada al faro, despertando en las noches de tormenta para cumplir su promesa.  —Mi deber es eterno —dijo él, mientras su figura comenzaba a desvanecerse como la niebla—. Cuida de la luz, porque en la oscuridad se revelan sombras que solo un guardián puede ver.  Antes de que Lucía pudiera decir más, el farero desapareció, dejando solo el sonido del viento y las olas. Esa noche, Lucía entendió que el faro no era solo una torre de luz, sino también un vínculo con el pasado, con la promesa de un hombre que seguía protegiendo el mar, aun después de la muerte.  Desde entonces, cada vez que una tormenta se acercaba, Lucía subía al faro, sabiendo que aunque no volviera a ver al farero, su presencia siempre estaba allí, vigilando en las sombras.  **PREGUNTAS**   1. **¿Qué le dijo el farero a Lucía antes de desaparecer?** A) Que cuidara de la luz del faro B) Que huyera antes de que llegara la tormenta C) Que se marchara del pueblo para siempre. 2. **¿Qué sucedía con el farero durante las tormentas?** A) Su sombra despertaba para guiar a los barcos B) Se transformaba en una criatura del mar C) Aparecía para encender manualmente el faro. |

**ACTIVIDAD 28**

**¿Qué será?**

**Ejercicio 28.1**

**Instrucciones:** La figura que tienes frente a ti está dividida en secciones pequeñas, cada una numerada con un dígito específico. Cada número corresponde a un color particular. Colorea cada sección siguiendo el código de colores indicado. Asegúrate de prestar atención a los números para obtener el resultado deseado.

**1: AZUL 2: NEGRO 3: GRIS 4: CAFÉ**

**
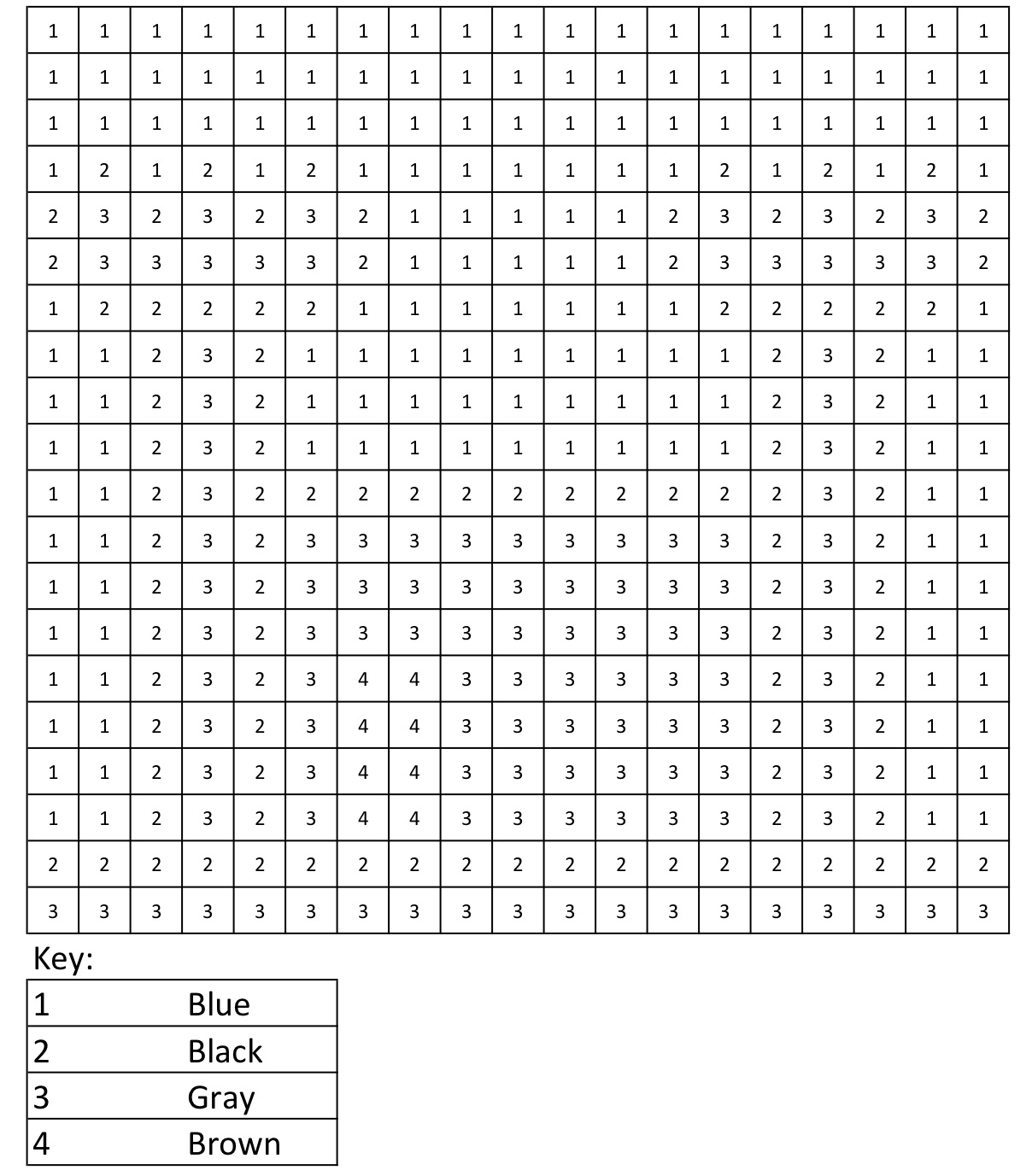
**
