# Supplementary material for: Cognitive Stimulation and Its Effects on Well-Being, Executive Functions, and Brain-Derived Neurotrophic Factor in Older Adults from a Mexican Geriatric Center: A Quasi-Experimental Study
Source: Nurs Rep. 2025 Apr 30;15(5):151. doi: 10.3390/nursrep15050151 (PMC12113664; doi:10.3390/nursrep15050151)
Supplement: Supplementary file 1 [file nursrep-15-00151-s001.zip › nursrep-3525323-Supplementary Material Table S1 STROBE-checklist.docx]

Table S1: STROBE Statement—checklist

|  | Item No. | Recommendation | Page  No. | Relevant text from manuscript |
| --- | --- | --- | --- | --- |
| **Title and abstract** | 1 | (*a*) Indicate the study’s design with a commonly used term in the title or the abstract | Page 1 (Title and Abstract) | Title includes 'A quasi-experimental study' and abstract describes design. |
|  |  | (*b*) Provide in the abstract an informative and balanced summary of what was done and what was found | Page 1 | Abstract summarizes intervention, assessments, and key findings. |
| Introduction | | | |  |
| Background/rationale | 2 | Explain the scientific background and rationale for the investigation being reported | Pages 2-3 | Introduction discusses aging, cognitive decline, and need for interventions. |
| Objectives | 3 | State specific objectives, including any prespecified hypotheses | Page 4 | Specific aims are presented in section '1.1 Aims'. |
| Methods | | | |  |
| Study design | 4 | Present key elements of study design early in the paper | Page 5 | Described in '2.1 Study Design' section. |
| Setting | 5 | Describe the setting, locations, and relevant dates, including periods of recruitment, exposure, follow-up, and data collection | Page 5 | Geriatric Center in Mexico; recruitment and intervention dates provided. |
| Participants | 6 | (*a*) *Cohort study*—Give the eligibility criteria, and the sources and methods of selection of participants. Describe methods of follow-up  *Case-control study*—Give the eligibility criteria, and the sources and methods of case ascertainment and control selection. Give the rationale for the choice of cases and controls  *Cross-sectional study*—Give the eligibility criteria, and the sources and methods of selection of participants | Page 5-6 | Inclusion/exclusion criteria, recruitment method described. |
|  |  |  |  |  |
| Variables | 7 | Clearly define all outcomes, exposures, predictors, potential confounders, and effect modifiers. Give diagnostic criteria, if applicable | Pages 6-9 | Outcomes: executive functions, BDNF, well-being; detailed measures. |
| Data sources/ measurement | 8* | For each variable of interest, give sources of data and details of methods of assessment (measurement). Describe comparability of assessment methods if there is more than one group | Page 3-4 | Data for well-being indicators were obtained from WHOQOL-OLD, Yesavage Geriatric Depression Scale (GDS-15) and Barthel Index. Cognitive assessment was evaluated by BANFE-3.  BDNF levels were measured using the ELISA technique. |
| Bias | 9 | Describe any efforts to address potential sources of bias | Page 5, 15 | Blinded assessors, standardized intervention, discussed in limitations. |
| Study size | 10 | Explain how the study size was arrived at | Page 5 | Sample size calculation described with statistical parameters. |

| Quantitative variables | | 11 | | Explain how quantitative variables were handled in the analyses. If applicable, describe which groupings were chosen and why | Page 10 | | Quantitative variables treated as continuous or categorical; groupings based on score ranges and clinical relevance. |
| --- | --- | --- | --- | --- | --- | --- | --- |
| Statistical methods | | 12 | | (*a*) Describe all statistical methods, including those used to control for confounding | Pages 10-11 | | Statistical analyses included t-tests, ANOVA, non-parametric tests; confounding addressed by stratification and group matching. |
|  |  |  |  | (*b*) Describe any methods used to examine subgroups and interactions | Page 11 | | Subgroup analyses performed between intervention and control groups with pre-post comparisons. |
|  |  |  |  | (*c*) Explain how missing data were addressed | Not applicable | | Not applicable |
|  |  |  |  | (*d*) *Cohort study*—If applicable, explain how loss to follow-up was addressed  *Case-control study*—If applicable, explain how matching of cases and controls was addressed  *Cross-sectional study*—If applicable, describe analytical methods taking account of sampling strategy | Not applicable | | Not applicable |
|  |  |  |  | (*e*) Describe any sensitivity analyses | Not applicable | | Not applicable |
| Results | | | | | | | |
| Participants | | 13* | | (a) Report numbers of individuals at each stage of study—eg numbers potentially eligible, examined for eligibility, confirmed eligible, included in the study, completing follow-up, and analysed | Page 11 | | Number of participants assigned, completed, and analysed provided in text. |
|  |  |  |  | (b) Give reasons for non-participation at each stage | Not applicable | | Not applicable |
|  |  |  |  | (c) Consider use of a flow diagram | Not included | | No flow diagram provided, though participant flow described in text. |
| Descriptive data | | 14* | | (a) Give characteristics of study participants (eg demographic, clinical, social) and information on exposures and potential confounders | Table 4, Page 12 | | Sociodemographic and baseline characteristics presented. |
|  |  |  |  | (b) Indicate number of participants with missing data for each variable of interest | Not applicable | | Not applicable |
|  |  |  |  | (c) *Cohort study*—Summarise follow-up time (eg, average and total amount) | Not applicable | | Not applicable |
| Outcome data | | 15* | | *Cohort study*—Report numbers of outcome events or summary measures over time | Not applicable | | Not applicable |
|  |  |  |  | *Case-control study—*Report numbers in each exposure category, or summary measures of exposure | Not applicable | | Not applicable |
|  |  |  |  | *Cross-sectional study—*Report numbers of outcome events or summary measures | Page 7 -12 | | Outcome events and summary measures are reported as means, proportions, or counts. |
| Main results | | 16 | | (*a*) Give unadjusted estimates and, if applicable, confounder-adjusted estimates and their precision (eg, 95% confidence interval). Make clear which confounders were adjusted for and why they were included | Page 10 | | Score ranges defined for categories of cognitive performance. |
|  |  |  |  | (*b*) Report category boundaries when continuous variables were categorized | Page 13-14 | | Subgroup analyses and interaction effects explored. |
| Other analyses | 17 | | Report other analyses done—eg analyses of subgroups and interactions, and sensitivity analyses | | Not applicable | Not applicable | |
| Discussion | | | | | | | |
| Key results | 18 | | Summarise key results with reference to study objectives | | Page 14 | Key results summarized at beginning of discussion. | |
| Limitations | 19 | | Discuss limitations of the study, taking into account sources of potential bias or imprecision. Discuss both direction and magnitude of any potential bias | | Pages 15-16 | Limitations include small sample size, lack of active control. | |
| Interpretation | 20 | | Give a cautious overall interpretation of results considering objectives, limitations, multiplicity of analyses, results from similar studies, and other relevant evidence | | Pages 14-15 | Interpretation consistent with objectives and literature. | |
| Generalisability | 21 | | Discuss the generalisability (external validity) of the study results | | Page 16 | External validity discussed; call for replication in diverse settings. | |
| Other information | | |  | | | | |
| Funding | 22 | | Give the source of funding and the role of the funders for the present study and, if applicable, for the original study on which the present article is based | | Page 17 | Funding acknowledged from IDEA GTO; funders had no role in study design or analysis. | |
